# Synthesis of PEDOT/CNTs Thermoelectric Thin Films with a High Power Factor

**DOI:** 10.3390/ma17051121

**Published:** 2024-02-29

**Authors:** Mohammad Ali Nasiri, Seong Yuen Tong, Chungyeon Cho, Clara M. Gómez, Andres Cantarero, Mario Culebras

**Affiliations:** 1 Institute of Molecular Science, University of Valencia, Carrer del Catedràtic José Beltrán 2, 46980 Valencia, Spain; nasiri@alumni.uv.es; 2 Materials Science Institute, University of Valencia, 46980 Paterna, Spain; tongse@alumni.uv.es (S.Y.T.); clara.gomez@uv.es (C.M.G.); 3 Department of Carbon Convergence Engineering, College of Engineering, Wonkwang University, Iksan 54538, Republic of Korea; cncho37@wku.ac.kr

**Keywords:** green synthesis, high thermoelectric materials, organic materials, doping states, flexible thin films, flexible thermoelectric materials, Raman spectroscopy, Hall effect

## Abstract

In this study, we have improved the power factor of conductive polymer nanocomposites by combining layer-by-layer assembly with electrochemical deposition to produce flexible thermoelectric materials based on PEDOT/carbon nanotubes (CNTs)—films. To produce films based on CNTs and PEDOT, a dual approach has been employed: (i) the layer-by-layer method has been utilized for constructing the CNTs layer and (ii) electrochemical polymerization has been used in the synthesis of the conducting polymer. Moreover, the thermoelectric properties were optimized by controlling the experimental conditions including the number of deposition cycles and electropolymerizing time. The electrical characterization of the samples was carried out by measuring the Seebeck voltage produced under a small temperature difference and by measuring the electrical conductivity using the four-point probe method. The resulting values of the Seebeck coefficient S and σ were used to determine the power factor. The structural and morphological analyses of CNTs/PEDOT samples were carried out using scanning electron microscopy (SEM) and Raman spectroscopy. The best power factor achieved was 131.1 (μWm^−1^K^−2^), a competitive value comparable to some inorganic thermoelectric materials. Since the synthesis of the CNT/PEDOT layers is rather simple and the ingredients used are relatively inexpensive and environmentally friendly, the proposed nanocomposites are a very interesting approach as an application for recycling heat waste.

## 1. Introduction

Nowadays, energy production is mainly based on non-renewable resources such as coal, oil, or natural gas. Pushed by developing countries, there is an increase in energy demand while our main energy resources are running out. Furthermore, the use of non-renewable resources results in an environmental catastrophe or greenhouse catastrophe. In the past few decades, a significant number of resources have been expended in the development of alternative energy resources compatible with the economic growth of human civilization. At the same time, the efficiency of the current energy production (based on fossil fuels) must be urgently improved since more than two-thirds of the total energy produced is wasted as heat, contributing to the greenhouse effect. Therefore, the conversion of waste heat into electricity is crucial for the sustainable development of our society. There are many technologies, all of them in progress, to recover waste heat [1] but only a few convert heat into electricity, with thermoelectricity being one of the most interesting options since a large spectral range of radiation can be encompassed. Thermoelectric materials can generate electricity from temperature gradients through the Seebeck effect [2].

Thermoelectric thin films play a crucial role in the advancement of flexible and high-performance thermoelectric devices (F-HTEDs). The quest for materials that can simultaneously offer exceptional thermoelectric performance and flexibility has led researchers to explore various options. In contrast to organic conducting polymers and organic/inorganic composites, which often exhibit higher flexibility but lower thermoelectric efficiency, the current focus of research predominantly revolves around enhancing the flexibility of high-performance inorganic materials. Notably, flexible inorganic thin films have been designed with the specific aim of maintaining their high thermoelectric performance while increasing their flexibility. This emphasis on flexibility arises from the increasing demand for F-HTEDs in wearable scenarios. Traditional Bi_2_Te_3_-based thermoelectric thin films, such as Ag-doped Bi_2_Te_3_ highly-oriented thin films, have garnered attention for their remarkable thermoelectric performance near room temperatures, boasting a ZT as high as 1.26 [3]. However, the inherent crystal structure of Bi_2_Te_3_ imparts poor flexibility and the relatively low natural abundance of tellurium (Te) contributes to cost inefficiency. Consequently, there is a pressing need for the development of alternative inorganic thermoelectric thin films that can overcome the flexibility limitations and the cost concerns associated with Bi_2_Te_3_-based materials [4].

Inorganic materials such as Bi_2_Te_3_, SiGe, PbTe, and SnSe have excellent thermoelectric properties [5,6]. However, their scarcity in the Earth [7], toxicity [8], high production cost, and poor processability do not make them the ideal option for practical applications in the future. On the other hand, there is an increasing interest in organic materials due to several advantages over inorganic ones; some of them are eco-friendly, like lignin [9], and have low production costs, a light weight, easy production process, high abundance, low thermal conductivity (crucial for a high figure of merit), and high electrical conductivity [10,11]. Poly(3,4-ethylenedeoxythiophene) (PEDOT) is one of the most popular organic thermoelectric materials that have been intensively used during the last few years [12,13,14,15]. However, one the main drawbacks of PEDOT is its low power factor (PF) compared to inorganic materials. Pristine PEDOT has a Seebeck coefficient of 15–18 μVK^−1^ and a PF (PF = σ S^2^) of 0.01 µWm^−1^K^−2^ [16]. Among the intensive amount of research carried out on conducting polymers, PEDOT stands as a focal point for synthetic methodologies and the enhancement of its thermoelectric properties. To facilitate its practical utilization, it is necessary to improve its PF [17]. Hence, the crux for achieving a superior PF in a thermoelectric material lies in optimizing both σ and S [18].

Recent studies have delved into various reducing agents to refine the Seebeck coefficient in PEDOT, yielding PFs of 161 and 153 μVK^−1^ with tetrakis(dimethylamino)ethylene (TDAE) and hydrazine, respectively [19]. In addition, in one of our previous works, we managed to increase the Seebeck coefficient of PEDOT, doping with 1-ethyl-3-methylimidazolium bis(trifluoromethylsulfonyl)imide (BTFMSI), from 14 to 42 μVK^−1^, using hydrazine as a reducing agent [20]. Also, there are other methods such as the deposition of tin oxide nanoparticles on PEDOT: PSS (1-ethyl-3-methylimidazolium bis(trifluoromethylsulfonyl)imide) layers as proposed by J. J. Dong et al. [21]. Furthermore, Z. Fan et al. [22] have reported the sequential post-treatment using acids and bases to optimize S and σ. Furthermore, S. Tu et al. improved the S and σ of PEDOT: PSS films using a combination of dimethyl sufoxide (DMSO) solvent doping and DMSO/salt post-treatment (de-doping) [23]. In alternative studies, introducing double-walled carbon nanotubes (DWNTs) and multi-walled carbon nanotubes (MWCNTs) into a material has shown promise in enhancing its thermoelectric properties [24,25]. The insertion of conductive organic and inorganic thermoelectric nanoparticles onto graphene/CNTs matrices facilitates a synergistic integration, harnessing the respective advantages inherent in both inorganic and organic materials [26]. I. Paulraj. et al. enhanced the PF using a post-treatment of ethylene glycol (EG) and zinc chloride [16]; T. Yemata. et al. have shown the enhancement of PF using a sequential treatment with trifluoroacetic acid [27]. However, the demand for pliable and conductive frameworks has spurred the development of polymer thermoelectric composites incorporating carbon structures.

PEDOT has attracted significant interest in the production of diverse electronic devices. Its inherent flexibility allows for the creation of flexible thermoelectric power generators and sensors, making it a suitable resource for developing power sources for wearable electronic devices [11,28]. Furthermore, the material holds promise across diverse applications, such as temperature sensors utilizing the Seebeck effect, foldable touch screens, e-skin technology, flexible sensors, and adaptable solar panels [29].

Enhancing the PF of organic thermoelectric materials can be performed intrinsically, manipulating the doping status of the thermoelectric material and, extrinsically, integrating nanostructured fillers such as CNTs and graphene into polymers. In their studies, N. Nandihalli et al. [30], S. Tu et al. [23], B. Zhang et al. [31], and other researchers have illustrated how optimizing the doping states of the material enhances both the PF and the figure of merit (ZT = PF T/κ, T being the temperature and κ the thermal conductivity) [32,33].

The electrical and thermal conductivity exhibited by CNTs is contingent upon various structural parameters, including their diameter, chirality, and defects. MWCNTs comprise multiple concentric layers of graphene, in contrast to single-walled carbon nanotubes (SWCNTs), which consist of a single layer. The elevated electrical conductivity of MWCNTs, attributed to the presence of multiple layers facilitating the enhanced electron transport pathways, is accompanied by a distinct heat conduction coefficient. Conversely, the superior Seebeck coefficient of SWCNTs, arising from their unique electronic structure and one-dimensional nature, is noteworthy. Significantly, investigating the concurrent impact of increased electrical conductivity and alterations in thermal conductivity in polymer thermoelectric composites is crucial. The synergistic interplay between these two thermoelectric composite properties, considering the PF, underscores the importance of a comprehensive examination [34,35,36].

Numerous studies, such as those carried out by G. P. Moriarty et al. [37] and N. Nandihalli et al. [30], have illustrated improvements in the PF when incorporating nanostructured fillers recognized for their excellent electrical conductivity. However, these investigations have primarily concentrated on employing double-walled carbon nanotubes (DWCNTs), MWCNTs, and graphene. This highlights the need to investigate the potential of SWCNTs, which are renowned for their superior Seebeck coefficient, in similar contexts [38,39]. Also, we investigated a composite material comprising CNTs and PEDOT, fabricated through a combination of the Layer-by-Layer (LbL) method for CNT deposition and electrochemical deposition for PEDOT. The choice of these specific fabrication techniques was driven by their unique advantages. The LbL method allowed for precise and controlled layering of CNTs, ensuring a uniform and tailored structure on the substrate [40]. Simultaneously, electrochemical deposition was employed for PEDOT to leverage its versatility in generating conductive polymer coatings with enhanced electrical properties. The combination of these two techniques aimed to synergistically enhance the overall conductivity and stability of the composite material [41]. This strategic approach, detailed in the revised introduction section, provides a rationale for utilizing the strengths of both methods to create a composite material with improved performance characteristics.

Hence, in this work, we tackle the optimization of PF using the combination of electrochemical polymerization of PEDOT and the addition of nanostructured filler using the LbL method. The purpose of the work is to study whether the combination of both methods can produce high PF. The path to optimize the thermoelectric properties of CNTs/PEDOT films will include the optimization of several parameters such as the type of CNTs (MWCNTs and SWCNTs), number of LbL cycles, and electrochemical polymerization time. The optimization resulted in a PF of 131 μWm^−1^K^−2^, which is comparable to typical values reported for some inorganic thermoelectric materials [25,27].

## 2. Materials and Methods

Acquisition: The chemicals, including Poly (diallyldimethylammonium chloride) (PDDA), sodium deoxycholate (DOC), 3,4-ethylenedioxythiophene (EDOT), 1-Butyl-1-methylpyrrolidinium bis(trifluoromethylsulfonyl)imide (BTFMSI), and acetonitrile, were purchased to Sigma Aldrich (Sigma Aldrich, Madrid, Spain). These chemicals were utilized in their original form. MWCNTs and SWCNTs were purchased from Nanostructured & Amorphous Materials, Inc. (Katy, TX, USA). The MWCNTs feature specifications are a 12–15 nm outer diameter, 4 nm inner wall diameter, and a length exceeding 1 μm, with a carbon content of ≥95 wt%. On the other hand, the SWCNTs exhibit specifications with a 1–2 nm diameter and a length ranging from 1 to 3 μm, also possessing a carbon content of ≥95 wt%.

Preparation of Substrate: Polyethylene terephthalate (PET) films, with a thickness of 180 μm (known as ST 505 by DuPont Teijin and purchased from Tekra Corp., New Berlin, WI, USA), were subjected to a cleaning process involving rinsing with deionized water, followed by ethanol and air-drying. Subsequently, the PET films underwent Corona treatment using a BD-20C Corona Treater from Electro-Technic Products Inc. (Chicago, IL, USA) to enhance the adhesion of the initial layer by oxidizing the polymer surface.

**Figure 1:**
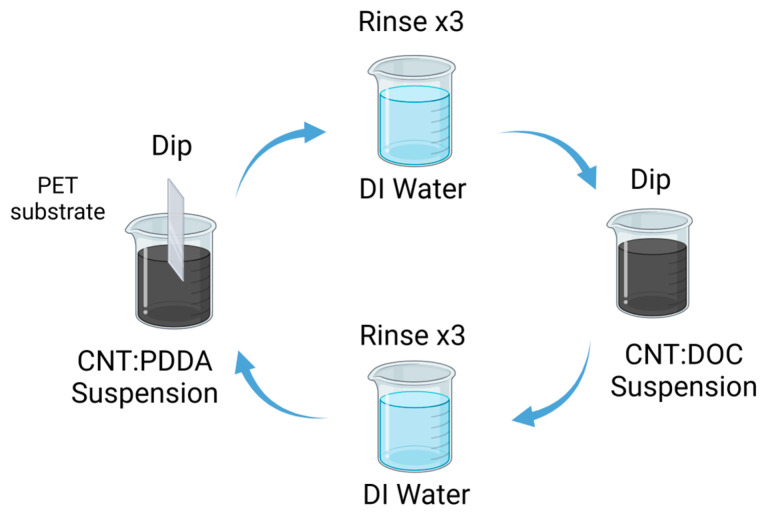
Schematic diagram of the LbL process for the preparation of the thermoelectric electrode [42].

Preparation of CNTs Films: A 0.05 wt% suspension of carbon nanotubes (MWCNTs or SWCNTs) was dispersed separately in aqueous solutions containing 0.25 wt% PDDA and 0.25 wt% DOC, respectively. The dispersion process involved weighing the necessary amounts of CNTs and PDDA, homogenizing the mixture and transferring it to a predetermined volume of deionized water. Similar steps were followed for the CNTs/DOC suspension. Both CNT suspensions underwent a sequence involving 15 min of stirring using an Ultra-Turrax disperser, followed by 15 min of tip sonication (70%, 1:0.1 s pulse) in an ice water bath and ultimately, 30 min of bath sonication to ensure homogeneity (see Figure 1). Each substrate was immersed first into the cationic PDDA-based suspension for 5 min, then rinsed in deionized water and air-dried. Subsequently, the substrate was immersed in the anionic DOC suspension for another 5 min. This procedure facilitated the deposition of a CNTs-PDDA/CNTs-DOC bilayer (BL). This cycle was repeated to achieve the desired number of bilayers and the films were left to air dry overnight.

Electrochemical Deposition of PEDOT: A solution comprising 0.01 M EDOT and 0.01 M BTFMSI in acetonitrile was prepared. Electrochemical polymerization of EDOT was conducted in a three-electrode cell at 3 mA using an Ag/AgCl reference electrode in an Ivium-n-Stat potentiostat (Ivium Technologies B.V., Eindhoven, The Netherlands) [43]. During the electrochemical polymerization process, a platinum grid was utilized as the counter electrode, with the CNTs film serving as the working electrode. Polymerization times of 0.5, 1, and 2 min were employed for the experiments.

Seebeck Measurement: A custom-designed system was constructed for the measurement of the Seebeck coefficient. This system comprised two copper blocks thermally linked by the films undergoing measurement. Two Peltier cells were employed to generate a controlled temperature gradient along the sample, facilitated by a Lakeshore 340 temperature controller (Lake Shore Cryotronics, Inc., Westerville, OH, USA). The system incorporated supplementary electronics to measure the voltage resulting from the thermal gradient, corresponding to the thermoelectric response of the sample. The Seebeck coefficient was calculated using the formula S = ∆V/∆T, where ∆V represents the voltage change due to the temperature difference ∆T [44]. All the measurements were averaged from 5 different samples. 

Electrical conductivity measurements: Electrical conductivity measurements were conducted utilizing a SIGNATONE Pro4 probe station, controlled by a Keithley 2400 multimeter (Keithley, Den Bosch, The Netherlands) through a LabView interface (NI LabVIEW 11.0) [44].

Hall effect measurements: Hall effect measurements were performed in the Van der Pauw geometry (10 mm × 10 mm) at room temperature using an Ecopia HMS-3000 measurement system (Ecopia, Anyang, Republic of Korea). To ensure repeatability and accuracy in determining carrier density, 20 separate measurements were made for each of these samples at three different currents (100, 150, and 200 µA) under a fixed magnetic field of 1 T. All the measurements were averaged from 5 different samples. 

Profilometry measurement: In the meticulous process of measuring the thickness of nanometric layers on samples, the Alpha-Step D-500 profilometry machine, manufactured by KLA Corporation (KLA, Milpitas, CA, USA), plays a pivotal role. The procedure begins with a deliberate scratch made on the sample surface using tools with a Rockwell hardness lower than that of the substrate, ensuring the avoidance of substrate scratching. Following this, the machine undergoes a thorough calibration process, utilizing standard samples provided by the company to guarantee precise measurements [45]. After calibration, the machine’s needle scans the surface, encompassing the scratched section of the sample. Following the scanning process, the machine’s software is utilized to analyze the generated graph. Initially, it is essential to identify the two sides of the scratch profile as aligned surfaces. Subsequently, the difference between these surfaces and the depth of the scratch is assessed, ultimately determining the thickness of the sample. Therefore, the Profilometer leverages its advanced technology to meticulously scan and analyze the surface profile, facilitating an accurate determination of the nanometric layer’s thickness. The integration of scratch creation and calibration with standard samples in this method ensures the reliability and precision of the thickness measurements obtained from the Alpha-Step D-500 Profilometer, establishing it as a key instrument in nanometric layer analysis.

Morphological characterization: Morphological characterization was conducted utilizing a Hitachi 4800 S field-emission scanning electron microscope (FE-SEM) (Hitachi High-Technologies Corporation, Tokyo, Japan) with an accelerating voltage of 20 kV and a working distance of 14 mm, specifically for surfaces coated with palladium-gold.

Raman Spectroscopy: Raman spectroscopy was conducted using a Horiba-MTB XploRA spectrometer (HORIBA Instruments Inc., Piscataway, NJ, USA) with an excitation wavelength of 514 nm. The Raman signal was recorded utilizing an open-electrode CCD detector within the range of 140–3060 cm^−1^, with an acquisition time of 50 s.

## 3. Result and Discussion

**Figure 2:**
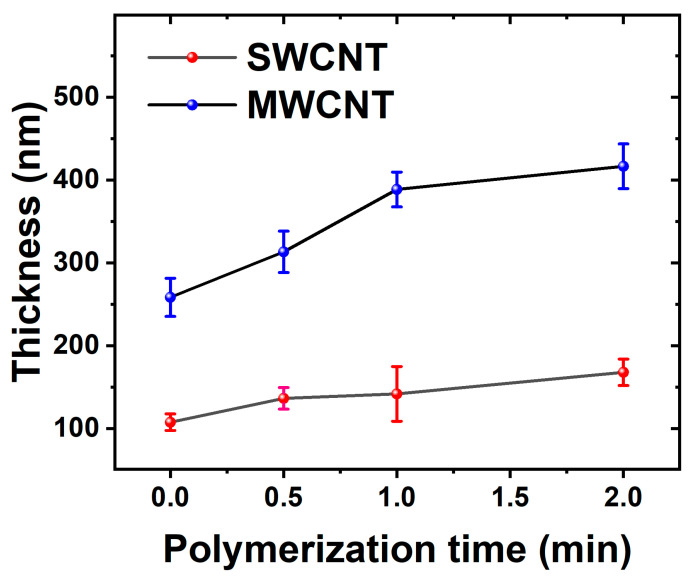
The electrode thickness evolution is depicted over the course of polymerization, utilizing electrodes based on MWCNTs and SWCNTs.

Both suspensions (SWCNTs and MWCNTs) showed good stability during the LbL deposition process, making it possible to produce very homogenous CTN films. For the subsequent electrochemical deposition of PEDOT, the number of cycles was selected to 30 based on preliminary optimization studies. Figure 2 shows the dynamic evolution of electrode thickness over time, with an initial rapid increase observed for both MWCNTs and SWCNTs electrodes, indicating an active polymerization phase during the early stages of deposition. As the process continues, the MWCNT electrode consistently maintains a higher thickness compared to the SWCNTs electrode, due to their bigger size.

The opposing impacts of electrical resistance on PEDOT polymerization in MWCNTs and SWCNTs derive from their unique structural and electrical characteristics. Initially, MWCNTs, featuring larger diameters and an extensive network, demonstrate higher electrical resistance during the early stages of deposition. However, as PEDOT polymerizes and integrates into the MWCNT network, it bridges individual nanotubes, reducing resistance and facilitating efficient electron propagation. This leads to the development of a more continuous and thicker PEDOT layer on the MWCNT electrode. Conversely, the smaller diameter and more compact structure of SWCNTs inherently yield lower electrical resistance, impeding the polymerization process and resulting in a comparatively thinner PEDOT layer on the SWCNT electrode [46].

**Figure 3:**
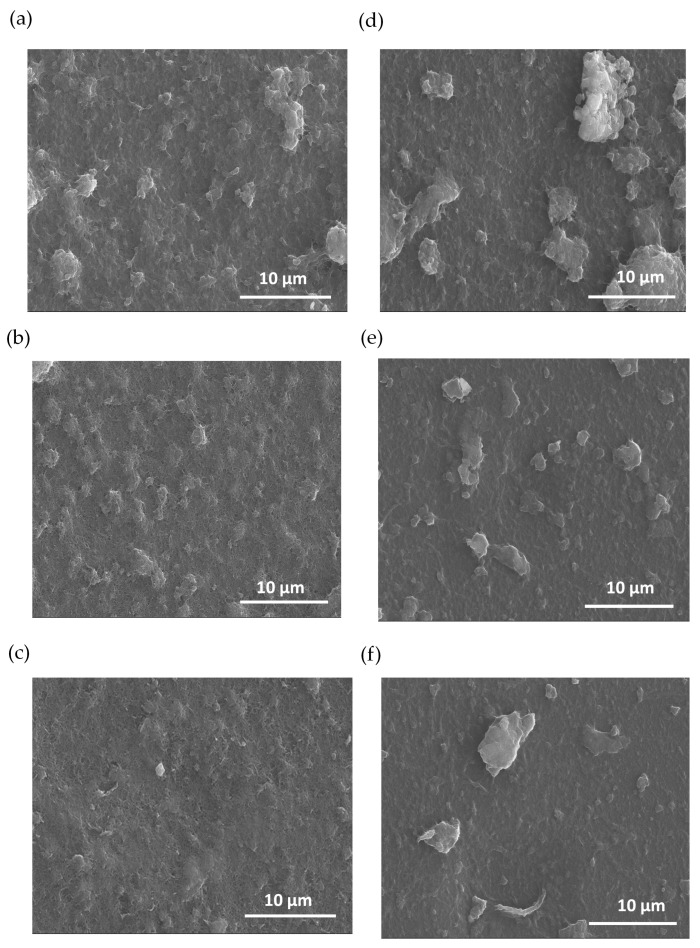
SEM images of carbon nanotube composites with PEDOT: BTFMSI at varying electrodeposition times (2, 1, and 0.5 min up to down), (**a**–**c**) MWCNT composition, and (**d**–**f**) SWCNT composition.

Figure 3 illustrates SEM images of PEDOT nanocomposites at various polymerization times. The morphological analysis indicates that there are changes in the films’ surface due to the electro-polymerization of PEDOT. The polymer is synthetized homogeneously on the CNT film, creating PEDOT domains that grow as a function of the polymerization time. In addition, there are notable differences between the initial electrodes based on MWCNTs and SWCNTs before polymerization. The images of Figure 3a–c pertain to the composites based on MWCNTs while the images of Figure 3d–f are for the films based on SWCNTs. Figure 3a–c show the morphology as a function of the PEDOT polymerization time, resulting in an expansion of PEDOT domains. For the case of the films based on SWNCTs (Figure 3d–f), the SEM images distinctly reveal regions characterized by agglomerates formed during the LbL deposition of a polymerized sample containing SWCNTs. This observation indicates that achieving dispersion is more challenging in samples polymerized with SWCNTs compared to those polymerized with MWCNT deposition, indicating that SWCNTs are more difficult to disperse compared to MWCNTs.

In addition, Figure 3 shows that the films composed of carbon nanotubes exhibit a notable absence of cracks after the polymerization of EDOT. The absence of cracks indicates that PEDOT is produced from the CNTs, filling the gaps between them resulting in a more compact material. Moreover, this effect helps to improve the electrical and thermoelectric properties of the final composite films [47].

SEM images of MWCNT and SWCNT samples, without the presence of PEDOT, are presented in a figure detailed in the Supplementary Materials. The images denote a higher roughness degree for the case of MWCNT films that can be attributed to their higher thickness compared to SWCNTs films. MWCNTs inherently contain multiple concentric layers contributing to higher film growth compared to SWCNTs. The sequential deposition of alternating layers, coupled with interactions between these layers and the substrate, contributes to a more ordered arrangement of MWCNTs on the substrate surface. This controlled layering and enhanced surface interaction led to the formation of thin films with uniform thickness, in contrast to SWCNTs which, being single-layered, may exhibit comparatively less homogeneity in the films produced using the same assembly method [48].

**Figure 4:**
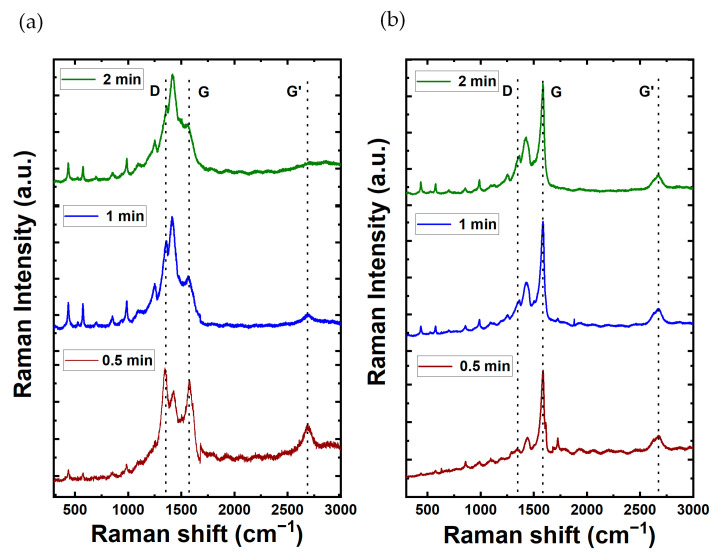
Raman spectra of carbon nanotubes composites with PEDOT at varying electrodeposition times (2, 1, and 0.5 min up to down) for (**a**) MWCNT and (**b**) SWCNT composition.

The Raman spectra for PEDOT composite films are shown in Figure 4. In addition, the Raman spectroscopy analysis for films comprising MWCNTs and SWCNTs in the absence of PEDOT is shown in Figure S2 from the SI file. The MWCNT spectrum reveals prominent characteristic peaks at 1300 cm^−1^ (D-band of graphene) and 1600 cm^−1^ (G-band), serving as key indicators of its distinct structural properties and configuration. This spectral insight serves to characterize and differentiate the structural features inherent in the MWCNT composition. Remarkably, the Raman spectra show distinctive features, each responsive to chiral indices (n, m) and characterized by the specification of the perimeter vector (chiral vector). The radial breathing mode (RBM) is discernible, wherein all carbon atoms move coherently in the radial direction. Additionally, the G-band represents neighboring atoms moving oppositely along the tube surface, akin to 2D graphite. The dispersive disorder-induced D-band and its second-order harmonic G′-band contribute further dimensions to the spectra. Among these features, the RBM emerges as particularly sensitive to the nanotube diameter (d_t_). For the case of SWCNT films, the G-band is located around 1580 cm^−1^ with a higher intensity compared to the D-band located around 1360 cm^−1^. The D and G band intensities ratio shows how good the bulk sample is and, if they are similar, it means there are more structural defects. MWCNTs have the lowest ratio, meaning more defects due to many graphite layers. On the other hand, SWCNTs, having only one wall, have more noticeable differences in D and G band intensities, indicating less structural defects. The G peak, which we observed at 1575.81 cm^−1^ for MWCNTs and 1585.32 cm^−1^ for SWCNTs, is indicative of the graphitic structure inherent in carbon nanotubes. In the case of SWCNTs, the D peak manifests at around 1337.54 cm^−1^, underscoring disorder or defects within the nanotube structure. Conversely, for MWCNTs, the D peak is detected in closed proximity to 1281.57 cm^−1^, signifying a comparable presence of structural irregularities. These spectroscopic findings highlight distinct characteristics in the graphitic structure and defects between MWCNTs and SWCNTs.

For the case of the CNTs/PEDOT films, the Raman spectra show peaks at 440.08, 576, and 990.82 cm^−1^, which corresponds to the deformation of oxyethylene ring. The peak of 689.77 cm^−1^ corresponds to the symmetric C-S-C deformation. The peak at 1100.28 cm^−1^ is related to C-O-C deformation. The 1261.84 and 1361 cm^−1^ correspond to C_α_–C_α_ (inter-ring) stretching and C_β_-C_β_ stretching, respectively. The symmetric C_α_=C_β_(–O) stretching mode is shown at 1430 cm^−1^. Finally, the bands at 1508 and 1568 cm^−1^ are both related to asymmetric C=C stretching. These peaks show that the material obtained in the synthesis is PEDOT. The peaks of MWCNTs (1300 cm^−1^ and 1600 cm^−1^) are not shown in the graph as the peaks were masked by the peaks of PEDOT [11]. Moreover, Table 1 provides a synopsis of the characteristic vibrational mode associated with the impact of PEDOT. Also, for the case of SWCNTs/PEDOT films, their spectra clearly show a sharp peak at around 1600 cm^−1^ which is attributable to the G-band of the SWNCTs [49].

**Figure 5:**
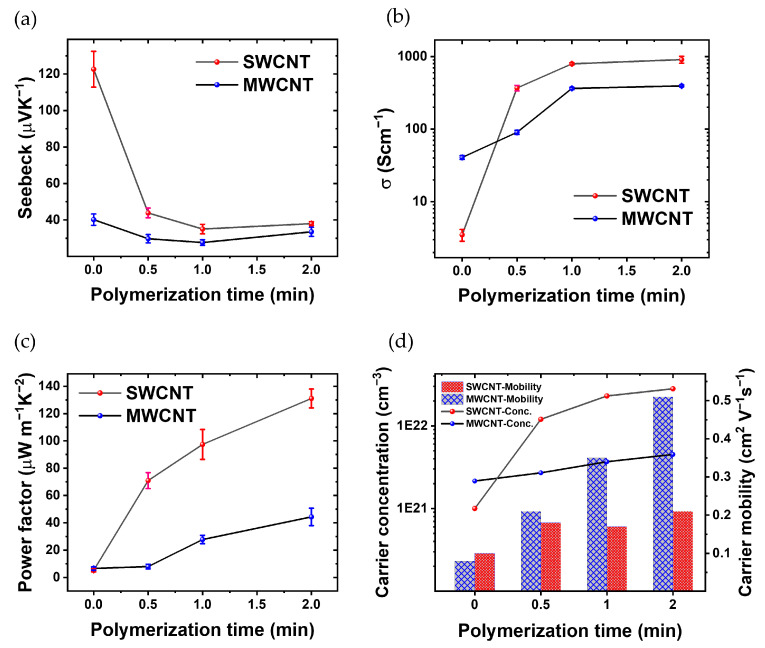
Thorough thermoelectric characterization, showcasing (**a**) the fluctuation in Seebeck Coefficient in relation to the polymerization time of PEDOT, (**b**) the changes in electrical conductivity across different polymerization times, (**c**) the dynamic variations in PF with respect to polymerization time, and (**d**) carrier concentration and carrier mobility of the CNTs/PEDOT films from Hall effect measurements.

Figure 5 show the thermoelectric characterization. Figure 5a shows the evolution of the Seebeck coefficient over the polymerization time for both MWCNTs and SWCNTs films. The Seebeck coefficient of SWCNTs experiences a consistent decline with increasing polymerization time. The Seebeck coefficient starts from 122 μVK^−1^ at t = 0; then, after polymerization reaches 37 μVK^−1^ (t = 2 min). In contrast, the Seebeck coefficient of MWCNTs exhibits a subtle reduction from t = 0 (no PEDOT) to t = 1 min, it starts at 40 μVK^−1^ and decreases to 33 μVK^−1^ after polymerization, remaining approx. within the same range after 1 min of PEDOT electrodeposition.

Figure 5b shows the electrical conductivity as a function of polymerization time. The films based on MWCNTs without PEDOT show a slightly higher electrical conductivity than SWCNTs. In the fabrication process, the primary electrode was developed using a LbL technique, incorporating 30 layers of MWCNTs. The electrical conductivity of this electrode is measured at 31.26 (Scm). This electrode plays a crucial role in the successive polymerization processes. Additionally, for SWCNTs within the same layer, the recorded electrical conductivity is 3.49 (Scm^−1^). With increasing the polymerization time, the conductivity of the composite with CNTs increases because PEDOT is the conductive polymer and thus the CNTs are linked electrically; the space between the CNT layers has been filled with a conductive polymer. Also, in the polymerization process of PEDOT: BTFMSI, MWCNTs and SWCNTs are employed as electrodes, imparting distinct advantages to the resulting composite material. The incorporation of these carbon nanotubes contributes to the enhancement of electrical conductivity. MWCNTs and SWCNTs, renowned for their intrinsic high electrical conductivity, establish conductive networks within the polymer matrix. The robust interconnection of CNTs facilitates efficient charge transport, creating pathways for electrons and holes. Moreover, the presence of MWCNTs and SWCNTs enhances charge carrier mobility, promoting effective electron transfer between PEDOT chains. The composite’s increased electrical conductivity is attributed to the synergistic effects of the CNTs, including their conductive nature, formation of interconnected pathways, and improved charge carrier mobility, all of which collectively contribute to the superior electrical properties of the PEDOT composite. Another hand, the electrical conductivity of the composite formed by polymerization with SWCNT electrodes surpasses that of the sample polymerized with MWCNTs electrodes, primarily due to the efficient interconnection of carbon nanotubes with the electrically conductive polymer PEDOT [50,51].

The results reveal a marked improvement in electrical conductivity upon the introduction of PEDOT. This effect is attributed to the inherent conductivity of PEDOT, which enhances the overall electrical performance of the material. These electrical conductivity disparities between SWCNTs-polymer and MWCNTs-polymer composites can be attributed to several influencing factors. Firstly, the distinct structural characteristics play a crucial role, as SWCNTs exhibit a one-dimensional arrangement of carbon atoms in a cylindrical structure, while MWCNTs possess multiple concentric layers. This unique structural difference allows SWCNTs to offer a more efficient pathway for electron transport compared to the more intricate structure of MWCNTs. In general, the variance in surface area per gram is noteworthy, with SWCNTs generally possessing a higher surface area per gram than MWCNTs, enhancing the interaction between SWCNTs and the polymer matrix, thereby contributing to an overall improvement in the conductivity of the composite [20,52]. Another contributing factor is the aspect ratio, where SWCNTs typically boast a higher length-to-diameter ratio than MWCNTs, facilitating a more effective percolation network within the polymer matrix and, consequently, enhancing electron transport and conductivity. Due to their smaller size and higher aspect ratio, SWCNTs exhibit enhanced dispersion, resulting in improved connectivity and conductivity within the composite.

The decrease in the Seebeck coefficient of SWCNTs (Figure 5a) cannot be completely attributed to the increase in the electrical conductivity (Figure 5b) since S decreases in a factor of three while σ increases three orders of magnitude. In the case of MWCNTs, S remains relatively constant throughout the polymerization process (Figure 5a), the reason could be the fact that the MWCNTs have an S comparable to that of pristine PEDOT [53]. In spite of that, σ increases up to two orders of magnitude with 2 min of polymerization.

Figure 5c shows the evolution of the PF as a function of PEDOT polymerization time. Initially, the thermoelectric composite incorporating SWCNTs demonstrates a superior PF at t = 0, 0.5, 1, and 2 min. Similarly, the PF of the thermoelectric composite incorporating MWCNTs increases as polymerization progresses. Consequently, the PF of the thermoelectric composite comprising PEDOT with SWCNTs is ~3 times higher than that of the composite incorporating MWCNTs after 2 min of polymerization. These results show that there is some room to improve the PF since S and σ are not completely dependent.

Hall effect measurements were conducted in the van der Pauw geometry to assess the electrical characteristics of SWCNT-polymer and MWCNT-polymer composites. Determination of the carrier concentration (*n*) and carrier mobility (*μ*) was achieved through the application of magnetic fields perpendicular to the electric current, inducing a Hall voltage for analysis. The reported values of carrier concentration and mobility represent averages from three independent measurements, ensuring robust and reproducible results. These parameters, along with the relationship between electrical conductivity (*σ*), *n*, and *μ*, as described by the formula *σ* = *neμ*, where *e* is the elementary charge, provide crucial insights into the materials’ electrical performance. This comprehensive analysis enhances the understanding of the thermoelectric behavior.

Figure 5d illustrates the evolution of critical thermoelectric parameters in the SWCNTs-polymer and MWCNTs-polymer composite as a function of polymerization time. As the polymerization time increases, a noteworthy trend emerges, showcasing a simultaneous rise in *σ* and *n*. This phenomenon is visually evident through the ascending curves, indicating an augmentation in the material’s ability to conduct electricity and carry charge. Furthermore, the *μ* is depicted in Figure 5d, revealing its dependence on polymerization time. The graph distinctly portrays the carrier mobility of the SWCNT-polymer composite, demonstrating an initial superiority over MWCNTs-polymer.

According to Figure 5d, before polymerization, SWCNTs exhibit a lower carrier concentration of 1 × 10^21^ (cm^−3^) compared to MWCNTs’ 2.15 × 10^21^ (cm^−3^). However, within 0.5 min of polymerization, SWCNTs’ carrier concentration jumps to 1.20 × 10^22^ (cm^−3^) and further escalates to 2.81 × 10^22^ (cm^−3^) after 2 min. In contrast, MWCNTs reaches 2.70 × 10^21^ (cm^−3^) after 0.5 min and 4.51 × 10^21^ (cm^−3^) after 2 min. Consequently, post-polymerization, the carrier concentration of SWCNTs is 6.2 times higher than that of MWCNTs. Additionally, the initial carrier mobility of SWCNTs (0.1 cm^2^V^−1^s^−1^) exceeds that of MWCNTs (0.08 cm^2^V^−1^s^−1^). After 0.5 min, MWCNTs’ carrier mobility increases to 0.21 cm^2^V^−1^s^−1^ (reaching 0.51 cm^2^V^−1^s^−1^ after 2 min), while SWCNTs achieve 0.18 cm^2^V^−1^s^−1^ (increasing to 0.21 cm^2^V^−1^s^−1^ after 2 min). As a result, post-polymerization, the carrier mobility of MWCNT surpasses that of SWCNT by 2.42 times. The comparative analysis of carrier concentration and mobility in Figure 5d offers valuable insights into the distinctive behavior of SWCNT-polymers as the polymerization times vary. This graphical representation acts as a visual aid, reinforcing the observed trends and relationships discussed in the accompanying text, thereby enhancing our comprehensive understanding of the material’s thermoelectric performance [25,54].

The LbL technique, relying on the electrostatic attraction between oppositely charged species, facilitated precise control over CNT thickness at the nanometer scale by adjusting the number of bilayers. Electrochemical deposition further enabled manipulation of the PEDOT layer thickness by varying the deposition time. The combination of both methods enabled a systematic exploration of their thermoelectric properties, allowing for a comprehensive study across varying BL numbers and deposition times [55]. The enhanced conductivity of the SWCNT electrode stems from its distinctive structural characteristics, characterized by superior electron transport properties. These attributes play a pivotal role in facilitating effective charge transfer during the polymerization process, resulting in the formation of a denser and thicker polymer layer on the electrode surface [56].

Therefore, the presented results highlight the critical role of electrode material conductivity in influencing the thickness of polymer layers during electrodeposition. The observed trend in Figure 5c emphasizes the superior performance of SWCNTs over MWCNTs in facilitating a more substantial and uniform polymer deposition. This understanding contributes to ongoing efforts to optimize electrode materials for enhanced electrochemical applications, providing valuable insights for the design and development of advanced composite materials. The chosen method for producing CNTs/PEDOT nanocomposites proved effective in generating highly homogeneous samples.

Table 2 presents a comprehensive overview of various methodologies utilized for enhancing the thermoelectric characteristics of materials. These methods use a range of strategies, including but not limited to optimizing doping levels, integrating nanostructured fillers (CNTs and SnO), and exploring novel material compositions. Each approach detailed in Table 2 encapsulates diverse experimental techniques and their respective outcomes, providing a broad landscape of techniques aimed at increasing the efficiency of thermoelectric materials. Looking at the results outlined in Table 2, our study showed very promising PF values being one the highest compared to similar carbon-based materials. Moreover, the use of SWCNTs reduces the PEDOT polymerization to a couple of minutes to reach PF values higher than 100 μWm^−1^K^−2^.

## 4. Conclusions

In this work, we have successfully enhanced the PF of a conductive polymer through a sustainable approach, aiming to boost the efficiency of a flexible thermoelectric module constructed from PEDOT-infused CNTs-based films. The synthesis of effective thermoelectric nanocomposites involved a strategic combination of the LbL method for the CNT layer formation and electrochemical polymerization for the synthesis of the conducting polymer, emphasizing the control over conductivity and doping with the incorporation of CNTs. The comprehensive characterization of the samples was conducted by measuring the Seebeck coefficient and assessing the electrical conductivity. These measured values of S and σ were then utilized to determine the PF. Remarkably, the combination of PEDOT and SWCNTs led the achievement of a competitive PF of 131.1 μWm^−1^K^−2^, rivaling traditional inorganic thermoelectric materials. One of the notable advantages of this synthesis approach lies in its simplicity, cost-effectiveness, and environmental friendliness, positioning it as a promising option for the recycling of heat waste.

## Figures and Tables

**Table 1:** Typical vibrational mode of PEDOT.

Raman Shift (cm^−1^)	Assignation
440.08	Oxyethylene ring def
575.99	Oxyethylene ring def
689.77	Symm C-S-C def
990.82	Oxyethylene ring def
1100.28	C-O-C def
1261.84	Cα–Cα (inter-ring) str
1361.00	Cβ-Cβ str
1429.04	Sym Cα=Cβ(–O) str
1508–1568	Asym C=C str

**Table 2:** The thermoelectric properties of different treatments of PEDOT.

Treatment	S (μVK^−1^)	σ (Scm^−1^)	PF (μWm^−1^K^−2^)	Reference
Chemical reduction with TDAE	161	-	-	[19]
Doping with BTFMSI and reduction with Hydrazine	42	708	147	[20]
Doping with DMSO and deposition of SnO NP	46.1	575	116	[21]
Treatment with H_2_SO_4_ then with NaOH	39.2	2170	334	[22]
Doping with DMSO and DMSO/salt post treatment	36	>800	105.2	[23]
Formation of nanocomposite using MWCNT	37–38	>2000	155	[25]
Post treatment with EG/ZnCl	24.83	1932	119	[16]
Sequential treatment with trifluoroacetic acid	17.5	3748	97.1	[27]
SWCNTs/PEDOT: BTFMSI	37.9	910.1	131.1	This work

## Data Availability

Data are contained within the article.

## Supplementary Materials

The following supporting information can be downloaded at https://www.mdpi.com/article/10.3390/ma17051121/s1. Figure S1: The SEM images of the CNTs samples; Figure S2: The RAMAN spectra of the CNTs films. Reference [57] is cited in the supplementary materials
