# Supplementary material for: Synthesis of PEDOT/CNTs Thermoelectric Thin Films with a High Power Factor
Source: Materials (Basel). 2024 Feb 29;17(5):1121. doi: 10.3390/ma17051121 (PMC10934363; doi:10.3390/ma17051121)
Supplement: Supplementary file 1 [file materials-17-01121-s001.zip › materials-2846023-supplementary.pdf]

materials

MDPI

Supporting Information

Synthesis of PEDOT/CNTs Thermoelectric Thin Films with a High Power Factor

Mohammad Ali Nasiri [1], Seong Yuen Tong [2], Chungyeon Cho [3], Clara M. Gómez [2], Andres Cantarero [1,*] and Mario Culebras [2,*]

[1] Institute of Molecular Science, University of Valencia, Carrer del Catedràtic José Beltrán 2, 46980 Valencia, Spain; nasiri@alumni.uv.es
[2] Materials Science Institute, University of Valencia, 46980 Paterna, Spain; tongse@alumni.uv.es (S.Y.T.); clara.gomez@uv.es (C.M.G.)
[3] Department of Carbon Convergence Engineering, College of Engineering, Wonkwang University, Iksan 54538, Republic of Korea; cncho37@wku.ac.kr
* Correspondence: andres.cantarero@uv.es (A.C.); mario.culebras@uv.es (M.C.)

Figure S1 showcases the SEM images capturing the structural characteristics of both multi-walled carbon nanotube (MWCNTs) and single-walled carbon nanotube (SWCNTs) samples in the absence of PEDOT. The images distinctly illustrate a higher level of islands in the MWCNTs sample compared to the SWCNTs sample. Consequently, in the absence of PEDOT, the SWCNTs sample exhibits a more homogenous structure in comparison to the MWCNTs sample [3]

(a) (b)

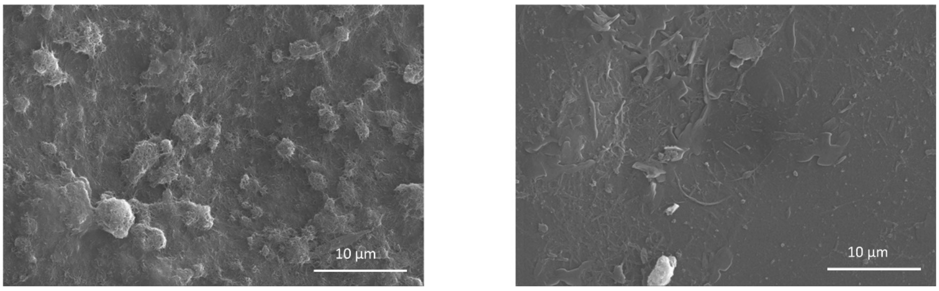

Figure S1. The SEM images of the CNTs samples. (**a**) MWCNTs. (**b**) SWCNTs.

Figure S2 presents the RAMAN spectra capturing the distinctive spectral profiles of both MWCNTs and SWCNTs films in the absence of PEDOT. The MWCNTs spectrum demonstrates characteristic peaks at 1300cm^{-1} and 1600cm^{-1}, indicative of its structural properties. Conversely, the RAMAN spectrum of SWCNTs distinctly exhibits a sharp and singular peak at 1600cm^{-1}, underscoring its unique spectral signature [1,2].

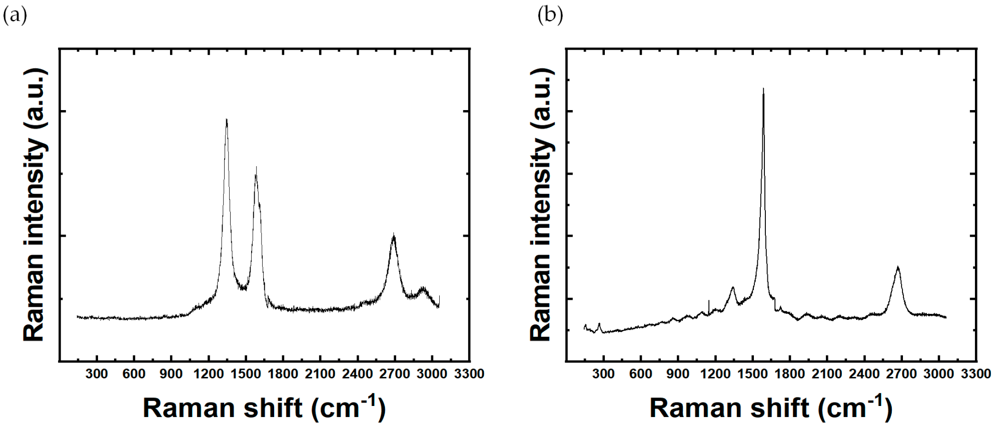

Figure S2. The RAMAN spectra of the CNT films. (**a**) MWCNT. (**b**) SWCNT.

References

1. Serrano-Claumarchirant, J.F.; Nasiri, M.A.; Cho, C.; Cantarero, A.; Culebras, M.; Gómez, C.M. Textile-based Thermoelectric Generator Produced Via Electrochemical Polymerization. *Adv. Mater. Interfaces* **2023**, *10*, 202202105. https://doi.org/10.1002/admi.202202105.
2. Costa, S.; Borowiak-Palen, E.; Kruszyńska, M.; Bachmatiuk, A.; Kaleńczuk, R.J. Characterization of carbon nanotubes by Raman spectroscopy. *Mater. Sci.* **2008**, *26*, 433–441.
3. Yusupov, K.; Zakhidov, A.; You, S.; Stumpf, S.; Martinez, P.; Ishteev, A.; Vomiero, A.; Khovaylo, V.; Schubert, U. Influence of oriented CNT forest on thermoelectric properties of polymer-based materials. *J. Alloys Compd.* **2018**, *741*, 392–397. https://doi.org/10.1016/j.jallcom.2018.01.010.

Disclaimer/Publisher's Note: The statements, opinions and data contained in all publications are solely those of the individual author(s) and contributor(s) and not of MDPI and/or the editor(s). MDPI and/or the editor(s) disclaim responsibility for any injury to people or property resulting from any ideas, methods, instructions or products referred to in the content.
